# Essence (Early Symptomatic Syndromes Eliciting Neurodevelopmental Clinical Examinations) and Spectrum Disorders - a common core?|

**DOI:** 10.1192/j.eurpsy.2023.1510

**Published:** 2023-07-19

**Authors:** G. C. Lima, T. C. Rocha, J. R. A. Leal

**Affiliations:** PSYCHIATRY, CHBM, Lisbon, Portugal

## Abstract

**Introduction:**

The concept of *ESSENCE* was created to coin a set of clinical symptoms in early childhood , before the age of 5 to 6 years, that may be present in several specifically undefined disorders and that may be associated with the development of more specific psychiatric disorders in adolescence and adulthood, such as Autism Spectrum Disorder, Schizophrenia or Bipolar Disorder.Those symptoms may include deficits in development, communication, language, social skills, motor coordination, attention, behavior, mood and sleep.

**Objectives:**

To evaluate the association between Neurodevelopmental Disorders, which manifest by uncharacteristic and diffuse symptoms in early childhood, and AutismSpectrum Disorder, Schizophrenia and Bipolar Disorder.

**Methods:**

We performed a non-systematic review of the existent literature with the keywords: “Attention Deficit”; “Hyperactivity Disorder”; “Autism Spectrum Disorder”; *“ESSENCE”*; “Schizophrenia”; and “Bipolar Disorder”.

**Results:**

Although *ESSENCE* is not a diagnostic term, some symptoms regarding ESSENCE are shared with early symptoms of different Major Psychiatric Disorders, namely speech and language delay, impulsivity, inattention, feeding difficulties, hypo/hyperactivity or other behavior problems.

There is a growing acceptance that the co-existence of disorders and the sharing of symptoms (so-called comorbidity) is a questionable concept, since we are usually not dealing with completely separate disorders.

Neurodevelopmental disorders present with frequent comorbidities and the overlap between the disordersstill needs to be better studied, as in autism spectrum disorder and attention deficithyperactivity, through a greater understanding of shared genetic and environmental factors and that reflect how early symptomatic syndromes can coexist in childhood, and later in adolescence and adulthood .

**Conclusions:**

The concept of ESSENCE emphasis the difficulty when making adiagnosis, specifically in Neurodevelopmental Disorders due to the fact that a variety osymptoms overlap. It is known that some disorders that will manifest in adulthood sharesymptoms with ESSENCE. Therefore, it is of great need to associate the current clinical findings with the present and future technologies, e.g. genetic markers, in order to dentify a common core with ESSENCE and Major Psychiatric Disorders.

**Disclosure of Interest:**

None Declared

